# Toxic wavelength of blue light changes as insects grow

**DOI:** 10.1371/journal.pone.0199266

**Published:** 2018-06-19

**Authors:** Kazuki Shibuya, Shun Onodera, Masatoshi Hori

**Affiliations:** Graduate School of Agricultural Science, Tohoku University, Sendai, Miyagi, Japan; University of Dayton, UNITED STATES

## Abstract

Short-wavelength visible light (blue light: 400–500 nm) has lethal effects on various insects, such as fruit flies, mosquitoes, and flour beetles. However, the most toxic wavelengths of blue light might differ across developmental stages. Here, we investigate how the toxicity of blue light changes with the developmental stages of an insect by irradiating *Drosophila melanogaster* with different wavelengths of blue light. Specifically, the lethal effect on eggs increased at shorter light wavelengths (i.e., toward 405 nm). In contrast, wavelengths from 405 to 466 nm had similar lethal effects on larvae. A wavelength of 466 nm had the strongest lethal effect on pupae; however, mortality declined as pupae grew. A wavelength of 417 nm was the most harmful to adults at low photon flux density, while 466 nm was the most harmful to adults at high photon flux density. These findings suggest that, as the morphology of *D*. *melanogaster* changes with growth, the most harmful wavelength also changes. In addition, our results indicated that reactive oxygen species influence the lethal effect of blue light. Our findings show that blue light irradiation could be used as an effective pest control method by adjusting the wavelength to target specific developmental stages.

## Introduction

The responses of insects to light have been studied and applied to various pest control methods. In particular, phototaxis has been well studied in insects [1–5]. By identifying the species of insects that are attracted to artificial light sources and counting the number of insects, it is possible to forecast pest outbreaks [6]. Phototaxis is also used for physical pest control. For instance, insects are attracted to light sources and are then killed by electric shock [7]. Another study found that the compound eyes of fruit-piercing moths are adapted to yellow light, which suppresses fruit-piercing behavior, with this information being used to control their behavior [8–10]. Furthermore, free-flying insects maintain their horizontal orientation by receiving sunlight on their dorsal side while flying, which is termed the dorsal light reaction [11]. This response has also been used in pest control. Specifically, by covering the ground in reflective mulching films (or sheets), flying insects receive reflected sunlight on the ventral side, which inhibits normal flight and prevents pests entering crop fields [12,13].

However, while many methods use light to control pests, these methods target the behavior of insects. While the lethal effects of ultra-violet light on insects have been reported over the last five decades [14–19], our previous study was the first, to our knowledge, to report the lethal effects of visible light on complex organisms, including insects [20]. Ultraviolet or blue light are used to attract insects to insect traps; however, our previous report suggested that insects are killed by continuous irradiation with blue light that is more than a certain amount. Depending on how it is used, blue light could be used to both capture insects in traps and remove pests through continuous irradiation. Perhaps, fluorescent lamps or LED, which are already used as light sources in insect traps, could be used as insecticidal devices. In our previous study, we irradiated the pupae of three species of insect with various wavelengths of blue light to determine the most harmful wavelength [20]. However, out of all insect species, the photosensitivity of insects that completely change the structure of the body via metamorphosis (termed holometabolism) might change, depending on the stage of development. Therefore, it is necessary to clarify the most toxic wavelength to each developmental (or growth) stage to establish new physical pest control techniques using blue light. In this study, we report how the effective wavelength of blue light changes with insect development. We selected the fruit fly, *Drosophila melanogaster*, as a model insect because of its short life cycle. Furthermore, because it is possible to rear large numbers of *D*. *melanogaster* with ease, the experiments of this study could be conducted efficiently. In addition, *D*. *melanogaster* is suitable for future research to clarify the mechanism that generates differences in the effective wavelength among developmental stages or species, because many mutants are produced and cell culture is straightforward.

## Materials and methods

### Insects

Wild type *D*. *melanogaster* individuals were purchased from Sumika Technoservice Co. (Takarazuka, Japan) for use in our experiments. The flies were the same strain as the flies used in our previous study (FBrf0227020) [20]. The flies were reared together in a plastic box (72 × 72 × 100 mm) on culture medium described by Hori et al [20]. The flies were maintained at 25 ± 1 °C under a light (L) to dark (D) photoperiod of 16L:8D in our laboratory.

### LED light radiation

LED lighting units (IS-mini^®^, ISL-150 × 150 Series, CCS Inc., Kyoto, Japan) and power supply units (ISC-201-2) were used for light radiation. Insects were irradiated with LED light in a multi-room incubator (LH-30CCFL-8CT; Nippon Medical & Chemical Instruments Co., Ltd., Osaka, Japan). The emission spectra (S1 Fig) and the number of photons were measured using a high-resolution spectrometer (HSU-100S; Asahi Spectra Co., Ltd., Tokyo, Japan; numerical aperture of the fiber: 0.2) in a dark room. During measurements, the distance between the LED lighting unit and the spectrometer sensor was set to be nearly the same as that between the insects and the LED lighting unit in the incubator.

### Lethal effects of blue light on *D*. *melanogaster* eggs

Five pairs of mated adult flies were released onto 10 ml culture medium (the same as the rearing culture medium) in a glass Petri dish (60 mm diameter × 90 mm tall). The females were allowed to lay 10 eggs on the medium within 6 h of being placed in the incubator (dark conditions, 25 ± 1 °C). Each Petri dish with 10 eggs was sealed with Parafilm and was placed in the incubator equipped with the LED lighting unit. The eggs were irradiated with different wavelengths of LED light for 48 h at 25 ± 1 °C. After irradiation, the number of newly hatched larvae was counted under a stereomicroscope. Considering the possibility of delayed hatching, the Petri dish was kept under dark conditions for three additional days after the initial count. Then, the number of hatching larvae was counted again. Lethal effects at 4.0 × 10^18^ photons· m^-2^·s^-1^ were compared among the six wavelengths (405, 417, 439, 454, 466, and 494 nm) and DD conditions. We also compared the relationships between the lethal effects and the number of photons among the six wavelengths. Ten replications (Petri dishes) were performed for each light dose and wavelength. The actual measurement value of the number of photons is shown in S1 Table.

### Lethal effects of blue light on *D*. *melanogaster* larvae

Ten final-instar larvae (wandering third-instar stage, L1 [21]) were collected from the plastic rearing boxes within 24 h of wandering out of the culture medium, and were placed in a polystyrene Petri dish (55 mm diameter × 15 mm tall). The Petri dish was sealed with Parafilm and was placed in the incubator equipped with the LED lighting unit. The larvae were irradiated with different wavelengths of light for 24 h at 25 ± 1 °C. After irradiation, the Petri dish was transferred to dark conditions and maintained for 10 days at 25 ± 1 °C. Then, the number of adults that emerged was counted. Lethal effects at 5.0 × 10^18^ photons·m^-2^·s^-1^ were compared among the six wavelengths (405, 417, 439, 454, 466, and 494 nm) and DD conditions. We also compared the relationships between lethal effects and the number of photons among the six wavelengths. Ten replications (Petri dishes) were performed for each light dose and wavelength. The actual measurement value of the number of photons is shown in S2 Table.

### Lethal effects of blue light on each developmental stage of *D*. *melanogaster* pupae

The pupae passed through 15 growth stages [21]. Photographs of each growth stage are shown in S2 Fig. To investigate the relationship between pupal growth and lethal effects, we assessed four pupal growth stages: P2–4, P5, P7–9, and P10–11. Each pupal stage was collected from the plastic rearing box under a stereomicroscope. Ten pupae were placed on a sheet of filter paper (Advantec, No. 1, 70 mm diameter) that had been impregnated with 250 μl water in each glass Petri dish (60 mm diameter × 20 mm tall). The Petri dish was sealed with Parafilm and was placed in the incubator equipped with the LED lighting unit. The pupae were irradiated with different wavelengths of light for 24 h at 25 ± 1 °C. After irradiation, the Petri dish was transferred to dark conditions and maintained for 9 days at 25 ± 1 °C. Then, the number of adults that emerged was counted. Lethal effects of irradiation at 8.0, 9.0, and 10.0 × 10^18^ photons·m^-2^·s^-1^ were compared among the six wavelengths (405, 417, 439, 454, 466, and 494 nm) and DD conditions. Ten replications (Petri dishes) were performed for each light dose and wavelength. The actual measurement value of the number of photons is shown in S3 Table.

### Lethal effects of blue light on *D*. *melanogaster* adults

Five pairs of adults were collected from the plastic rearing boxes within 12 h of emergence and were released onto 10 ml culture medium (the same as the rearing culture medium) in a glass Petri dish (60 mm diameter × 90 mm tall). The Petri dish was placed in the incubator equipped with the LED lighting unit. The adults were irradiated with different wavelengths of light for 12 days at 25 ± 1 °C. Every 3 days, we counted the number of dead adults and replaced the Petri dish containing the culture medium with a fresh one. Lethal effects of irradiation at 1.0, 5.0, and 10.0 × 10^18^ photons·m^-2^·s^-1^ were compared among the six wavelengths (405, 417, 439, 454, 466, and 494 nm) and DD conditions. Ten replications (Petri dishes) were performed for each light dose and wavelength. The actual measurement value of the number of photons is shown in S4 Table.

### H_2_O_2_ generation by irradiating blue light on *D*. *melanogaster* pupae

One pupa of P5(i)-25 stage [21] was collected from the plastic rearing boxes at 5 days after oviposition. The pupa was homogenized with 800 μl phosphate buffer solution and centrifuged (3000 rpm, 1 min). Supernatant was transferred to microtiter plate sealed with transparent film and irradiated with different wavelengths of light for 24 h at 25 ± 1 °C. The amount of H_2_O_2_ was measured by conversion to luminescence of luciferin using ROS-Glo^™^ H_2_O_2_ Assay (Promega Japan, Tokyo, Japan). The amount of luminescence was measured with luminometer (GloMax 20/ 20 Luminometer, Promega Japan, Tokyo, Japan) and represented with RLU (Relative Luminescence Unit). The RLU of irradiation at 1.0, 5.0, and 10.0 × 10^18^ photons·m^-2^·s^-1^ was compared among the six wavelengths (405, 417, 439, 454, 466, and 494 nm) and DD conditions. Thirty replications were performed for each light dose and wavelength. The actual measurement value of the number of photons is shown in S5 Table.

### Temperature measurements

The temperature of the inner space of the Petri dishes was measured using a button type temperature logger (Thermochron type-G, KN Laboratories, Inc., Osaka, Japan). The measurements were completed during irradiation with the strongest light intensity of each wavelength in each experiment. The temperature data of each developmental stage are summarized in S6–S9 Tables.

### Statistical analysis

Mortality was analyzed using the Steel-Dwass test. The RLU was analyzed using Tukey’s test. The calculations were performed using R version 3.0.3.

## Results

### Lethal effects of blue light on *D*. *melanogaster* eggs

**Fig 1:**
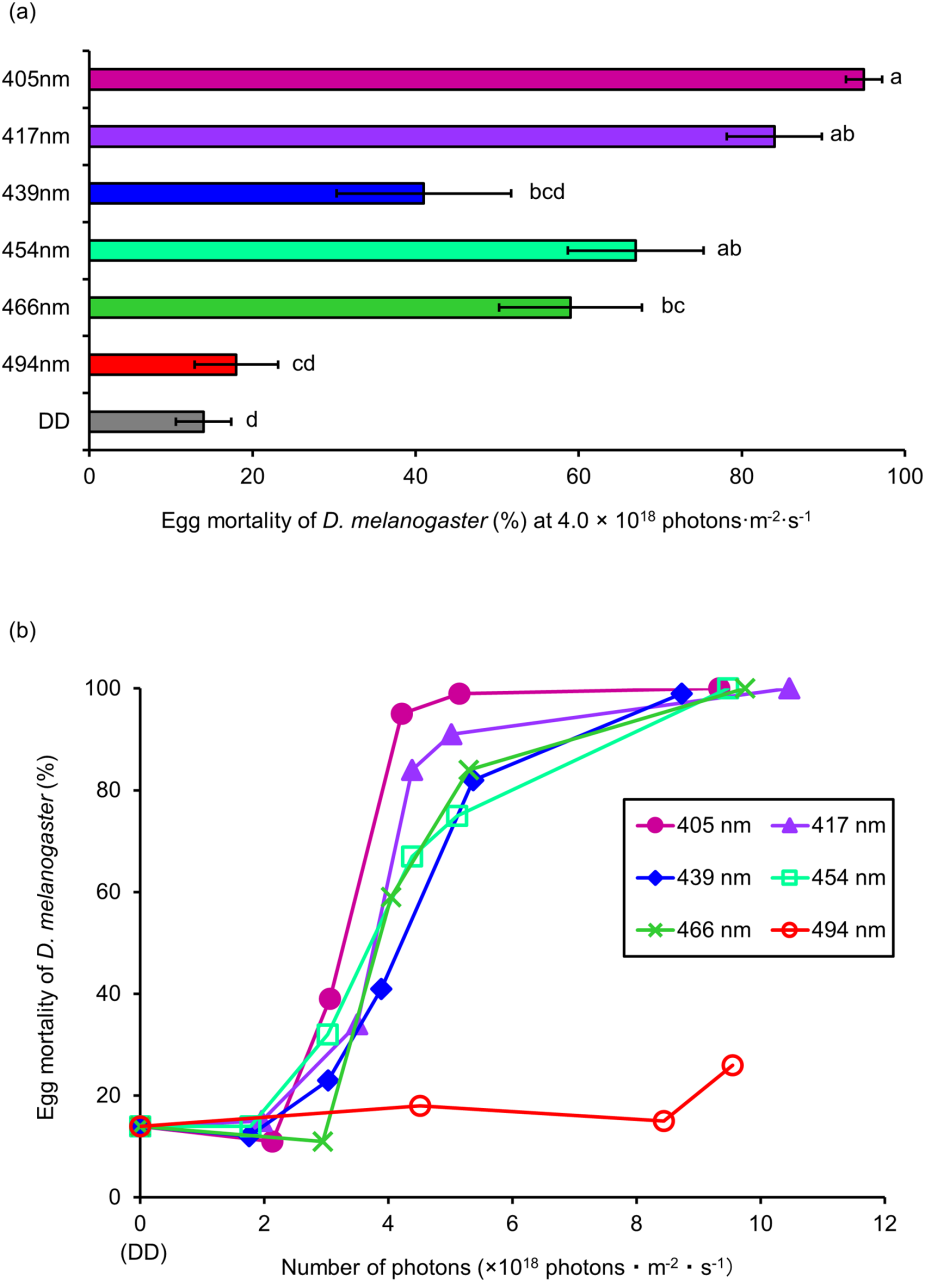
Lethal effect of different wavelengths of blue light on *D*. *melanogaster* eggs. (a) Mortality of eggs at 4.0 × 10^18^ photons·m^-2^·s^-1^ (mean ± standard error). Different lowercase letters next to bars indicate significant differences (Steel-Dwass test, *P* < 0.05). (b) Dose-response relationships for lethal effects (mean values). DD: 24 h dark conditions.

We investigated the lethal effect of different wavelengths of blue light (405, 417, 439, 454, 466, 494 nm) on *D*. *melanogaster* eggs. Ten replications were performed for each light dose and wavelength. In our previous study, while irradiation with 467 nm at 3.0 × 10^18^ photons·m^-2^·s^-1^ showed no lethal effects on egg stage, lethal effects were detected at > 4.0 × 10^18^ photons·m^-2^·s^-1^ [20]. Therefore, we irradiated the eggs initially at these light wavelengths at 4.0 × 10^18^ photons·m^-2^·s^-1^ (Fig 1a).

A wavelength of 405 nm (the shortest wavelength) had the most lethal effect, with 95% mortality. In contrast, 494 nm (the longest wavelength) showed the lowest lethal effect, with 18% mortality. The mortality of irradiated eggs tended to increase as the wavelength shortened, except for at 439 nm. Mortality at 439 nm was slightly lower than that at 417 and 454 nm. Next, we irradiated the eggs at the same wavelengths but with different intensities (i.e., different numbers of photons). For all wavelengths, mortality increased as the number of photons increased (Fig 1b). Mortality of 75–99% was recorded at 405 to 466 nm with 5.0 × 10^18^ photons·m^-2^·s^-1^. In contrast, mortality at 494 nm remained low (up to 26%), even when the number of photons was raised to 9.6 × 10^18^ photons·m^-2^·s^-1^.

### Lethal effects of blue light on *D*. *melanogaster* larvae

**Fig 2:**
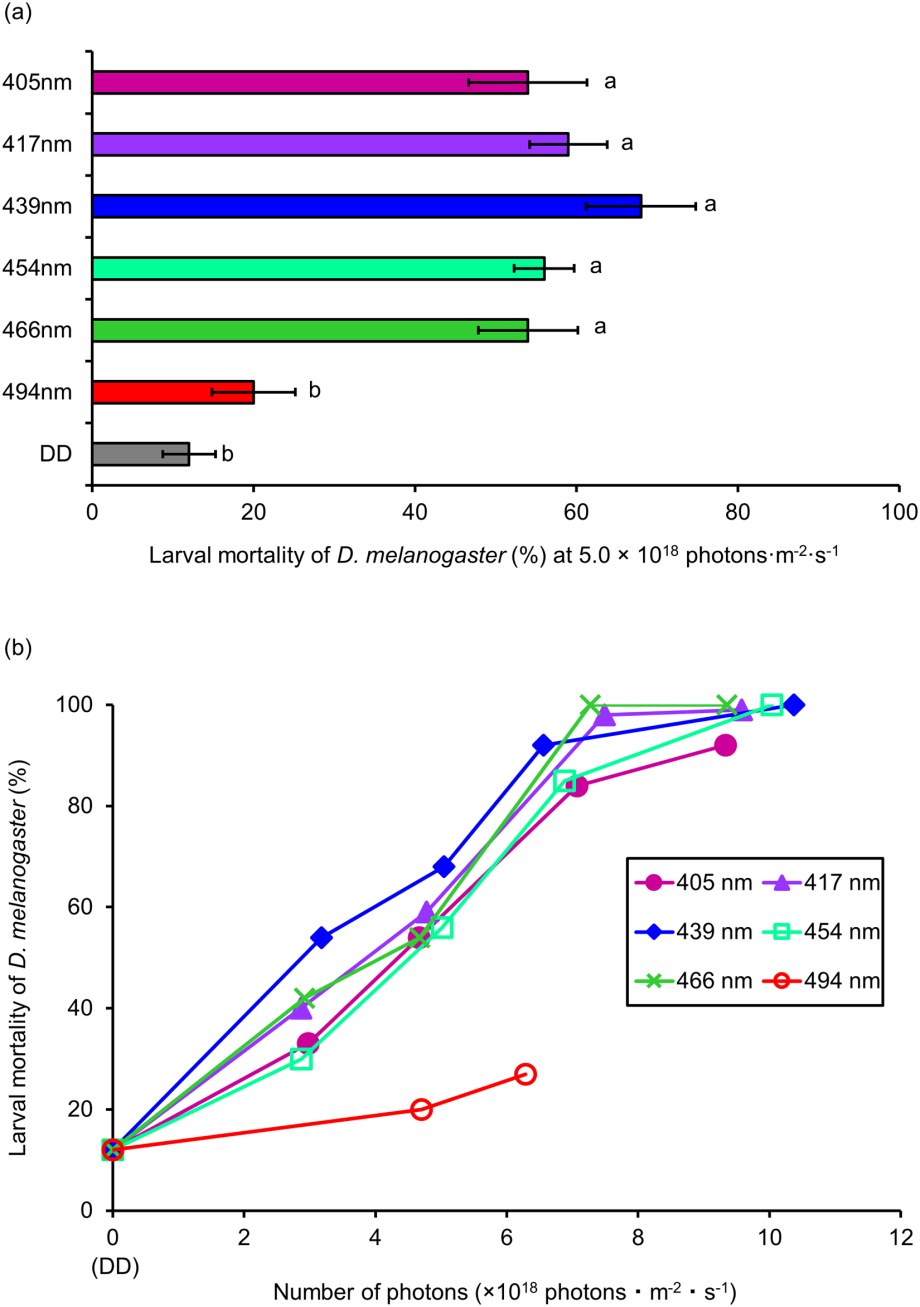
Lethal effect of different wavelengths of blue light on *D*. *melanogaster* larvae. (a) Mortality of larvae at 5.0 × 10^18^ photons·m^-2^·s^-1^ (mean ± standard error). Different lowercase letters next to bars indicate significant differences (Steel-Dwass test, *P* < 0.05). (b) Dose-response relationships for lethal effects (mean values). DD: 24 h-dark conditions.

Ten replications were performed for each light dose and wavelength. In our previous study, irradiation with 467 nm at 7.0 × 10^18^ photons·m^-2^·s^-1^ killed >90% of larvae before adult emergence, whereas, mortality at 5.0 × 10^18^ photons·m^-2^·s^-1^ was <60% [20]. It is difficult to compare to the lethal effects between wavelengths if the number of photons is over 7.0 × 10^18^ photons·m^-2^·s^-1^ because the mortality is too high. Therefore, we initially irradiated *D*. *melanogaster* larvae with different wavelengths of visible light (405, 417, 439, 454, 466, 494 nm) at 5.0 × 10^18^ photons·m^-2^·s^-1^ (Fig 2a).

Wavelengths from 405 to 466 nm showed similar lethal effects (54–68%), with mortality significantly differing to that of DD conditions (12%). In contrast, mortality at 494 nm was 20%, but was not significantly different to DD. Next, we irradiated larvae at several light intensities (Fig 2b). Mortality increased as the number of photons increased. For instance, when the number of photons was raised above 6.5 × 10^18^ photons·m^-2^·s^-1^, mortality at 405 to 466 nm exceeded 80%. In contrast, mortality at 494 nm with 6.3 × 10^18^ photons·m^-2^·s^-1^ was just 27%. The lethal effect of this wavelength was clearly lower than that of the other wavelengths.

### Lethal effects of blue light on each developmental stage of *D*. *melanogaster* pupae

**Fig 3:**
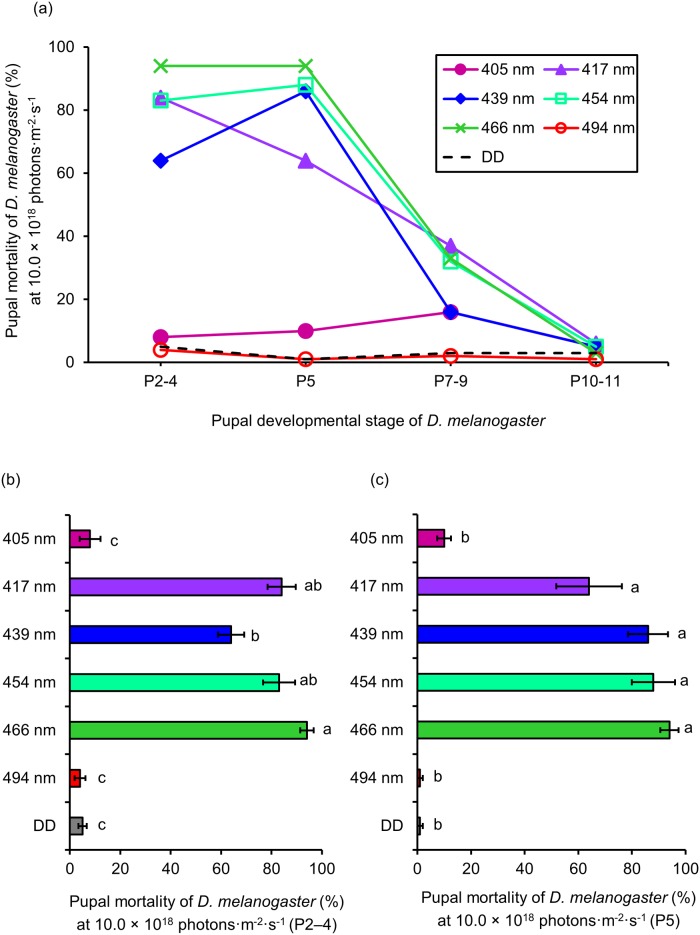
Lethal effect of different wavelengths of blue light on *D*. *melanogaster* pupae. (a) Mortality of each developmental stage (mean values) at 10.0 × 10^18^ photons·m^-2^·s^-1^. (b) Mortality of P2–4 and (c) P5 at 10.0 × 10^18^ photons·m^-2^·s^-1^ (mean ± standard error). Different lowercase letters next to bars indicate significant differences (Steel-Dwass test, *P* < 0.05). DD: 24 h dark conditions.

Ten replications were performed for each light dose and wavelength. According to Bainbridge & Bownes (1981) [21], pupal growth follows 15 phases (S2 Fig). Although we irradiated the pupae of *D*. *melanogaster* for 7 days in our previous study [20], to investigate how the lethal effect changes with pupal growth, we shortened irradiation period to 24 h. The number of photons was increased, rather than shortening irradiation period. Therefore, we irradiated four developmental stages (P2–4, P5, P7–9, P10–11) with various wavelengths of visible light (405, 417, 439, 454, 466, 494 nm) for 24 hours at 10.0 × 10^18^ photons·m^-2^·s^-1^ (Fig 3a).

Wavelengths of 417 to 466 nm had strong lethal effects (Fig 3a). Mortality was 64–94% for P2–4 and P5 at these wavelengths. However, mortality decreased with increasing pupal development. At the same wavelength range, mortality of P7–9 was 16–37%, while that of P10–11 was 3–6%. At 439 nm, mortality increased for P2–4 to P5. In contrast, wavelengths of 405 and 494 nm had no lethal effect on any growth stage. To compare differences in the lethal effects between wavelengths, multiple comparisons were carried out. Mortality of P2–4 was significantly higher at 417 to 466 nm than at the other wavelengths and under DD conditions. The wavelength with the highest mortality was 466 nm (94%), followed by 417 nm (84%), 454 nm (83%), and 439 nm (64%). Mortality at 405 and 494 nm was not significantly different to that under DD conditions (Fig 3b). Compared to other wavelengths and DD conditions, 417 to 466 nm showed significantly higher mortality for P5. The wavelength with the highest mortality for P5 was 466 nm (94%), followed by 454 nm (88%), 439 nm (86%), and 417 nm (64%). Mortality at 405 and 494 nm was not significantly different to that under DD conditions (Fig 3c).

**Fig 4:**
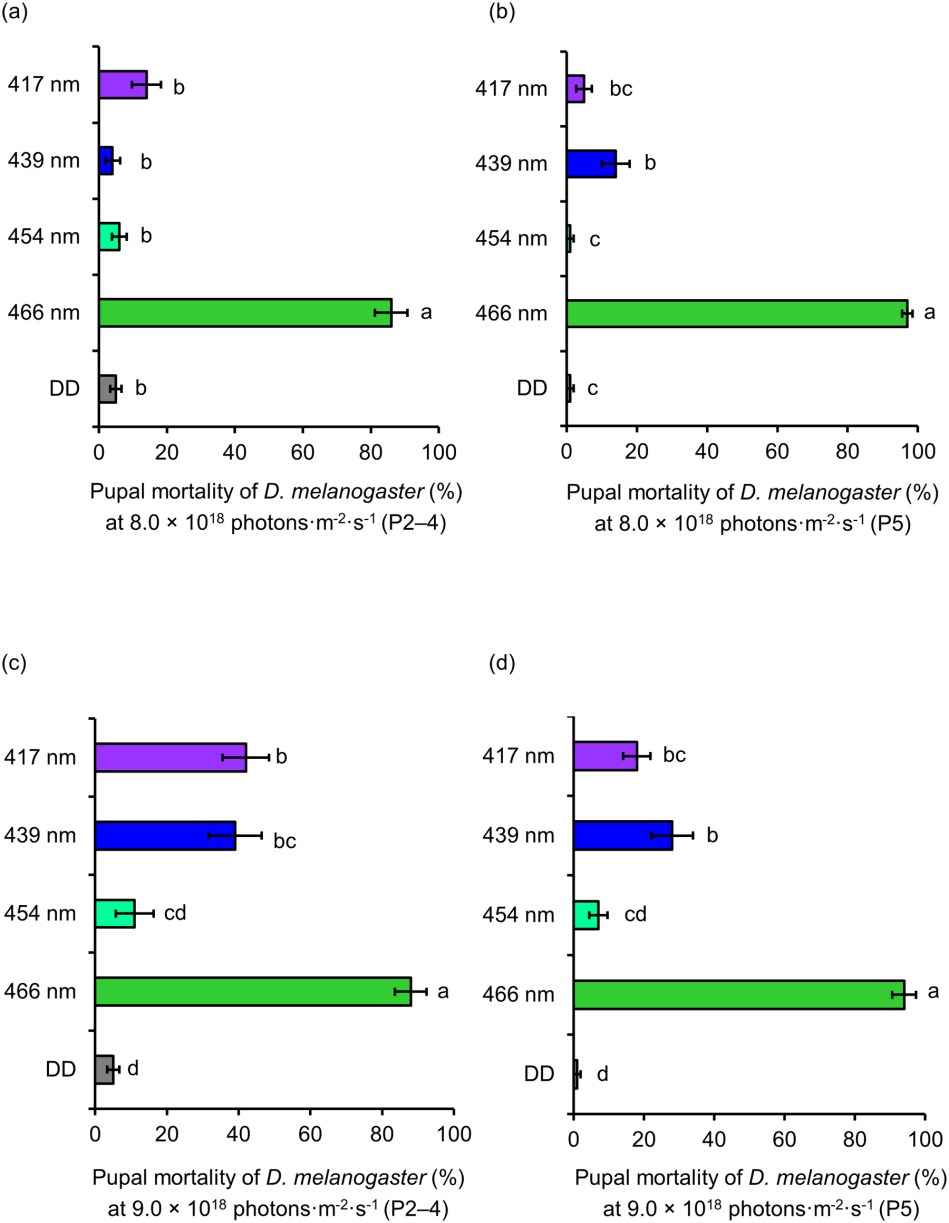
Lethal effect of different wavelengths of blue light on *D*. *melanogaster* pupae. (a) Mortality of P2–4 and (b) P5 at 8.0 × 10^18^ photons·m^-2^·s^-1^ (mean ± standard error). (c) Mortality of P2–4 and (d) P5 at 9.0 × 10^18^ photons·m^-2^·s^-1^ (mean ± standard error). Different lowercase letters next to bars indicate significant differences (Steel-Dwass test, *P* < 0.05). DD: 24 h dark conditions.

Similar irradiation experiments were conducted at wavelengths of 417 to 466 nm with 8.0 and 9.0 × 10^18^ photons·m^-2^·s^-1^, to investigate how the lethal effect changes with the number of photons (Fig 4).

When the number of photons was reduced to 8.0 × 10^18^ photons·m^-2^·s^-1^, high mortality was only detected at 466 nm for P2–4 (86%) and P5 (97%). Compared to DD, there was no significant difference in the mortality of P2–4 at 417, 439, and 454 nm (Fig 4a). Compared to DD, mortality of P5 was significantly different at 439 nm; however, mortality was just 14% (Fig 4b). The highest mortality of P2–4 (88%) and P5 (94%) was recorded at 466 nm with 9.0 × 10^18^ photons·m^-2^·s^-1^. Mortality of P2–4 at 417 and 439 nm was significantly different to DD. In contrast, mortality of P2–4 (11%) at 454 nm was not significantly different to DD (5%) (Fig 4c). Mortality of P5 at 417 and 439 nm was significantly different to DD, whereas mortality at 454 nm was similar to that under DD conditions (Fig 4d).

### Lethal effects of blue light on *D*. *melanogaster* adults

Ten replications were performed for each light dose and wavelength. In the experiment using adults, we irradiated different wavelengths of blue light for 12 days continuously, and we calculated the accumulated mortality every 3 days. In our previous study, irradiation with 467 nm at 1× 10^18^ photons·m^-2^·s^-1^ shortened adult longevity about one third [20]. In the current study, we initially irradiated *D*. *melanogaster* adults initially at 1× 10^18^ photons·m^-2^·s^-1^ because it was difficult to control the number of photons below 1× 10^18^ photons·m^-2^·s^-1^.

**Fig 5:**
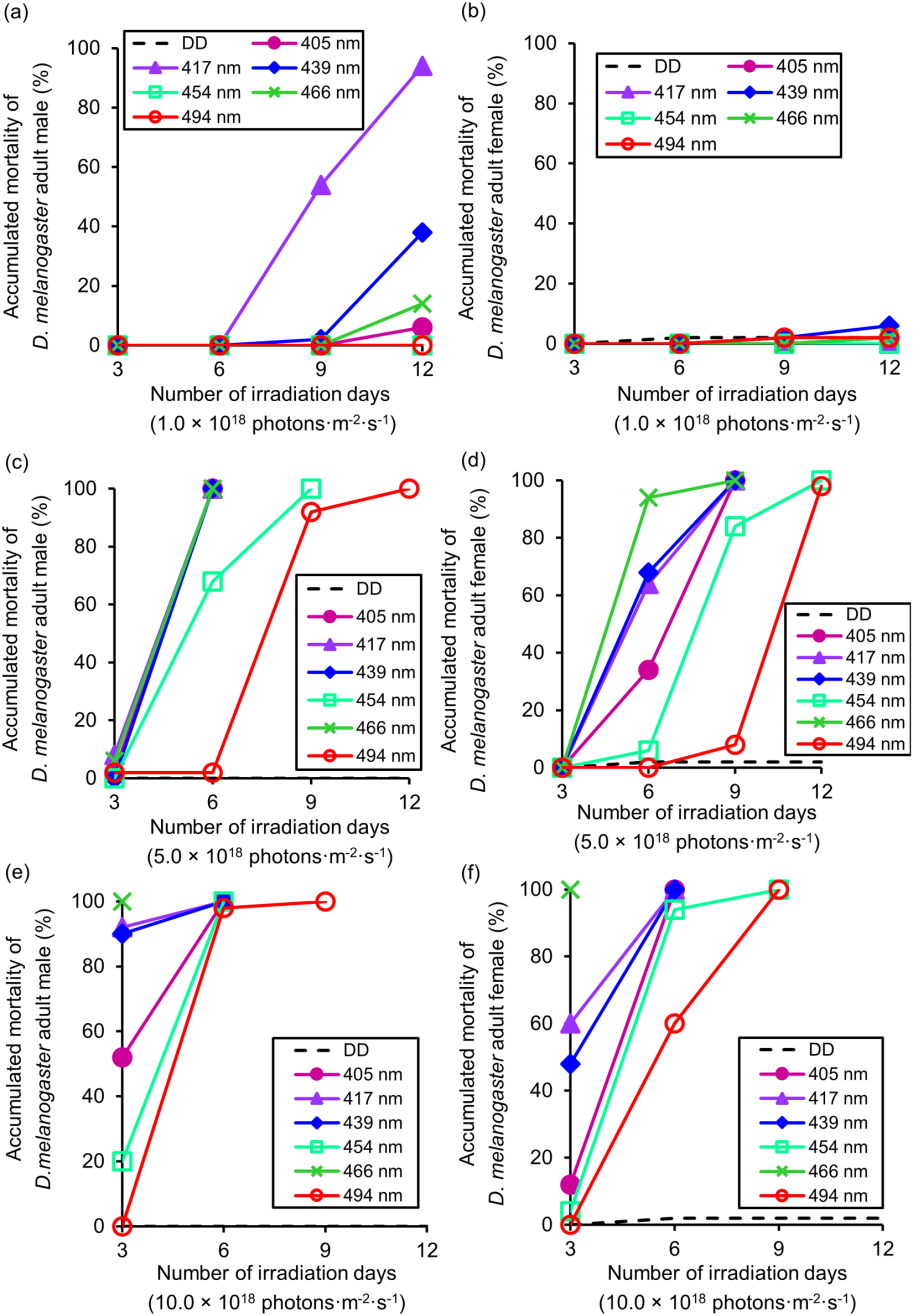
Lethal effect of different wavelengths of blue light on *D*. *melanogaster* adults. (a) Number of irradiation days versus accumulated male mortality at 1.0 × 10^18^ photons·m^-2^·s^-1^ (mean values). (b) Number of irradiation days versus accumulated female mortality at 1.0 × 10^18^ photons·m^-2^·s^-1^ (mean values). (c) Number of irradiation days versus accumulated male mortality at 5.0 × 10^18^ photons·m^-2^·s^-1^ (means). (d) Number of irradiation days versus accumulated female mortality at 5.0 × 10^18^ photons·m^-2^·s^-1^ (means). (e) Number of irradiation days versus accumulated male mortality at 10.0 × 10^18^ photons·m^-2^·s^-1^ (means). (f) Number of irradiation days versus accumulated female mortality at 10.0 × 10^18^ photons·m^-2^·s^-1^ (means).

At 1.0 × 10^18^ photons·m^-2^·s^-1^, none of the wavelengths showed any lethal effect on males for up to day 6 of irradiation. However, on day 9, mortality was 54% at 417 nm. On day 12, lethal effects were recorded at 439 nm (38% mortality) and 417 nm (94% mortality). No lethal effect was detected for any of the other wavelengths throughout the 12-day irradiation period (Fig 5a).

**Fig 6:**
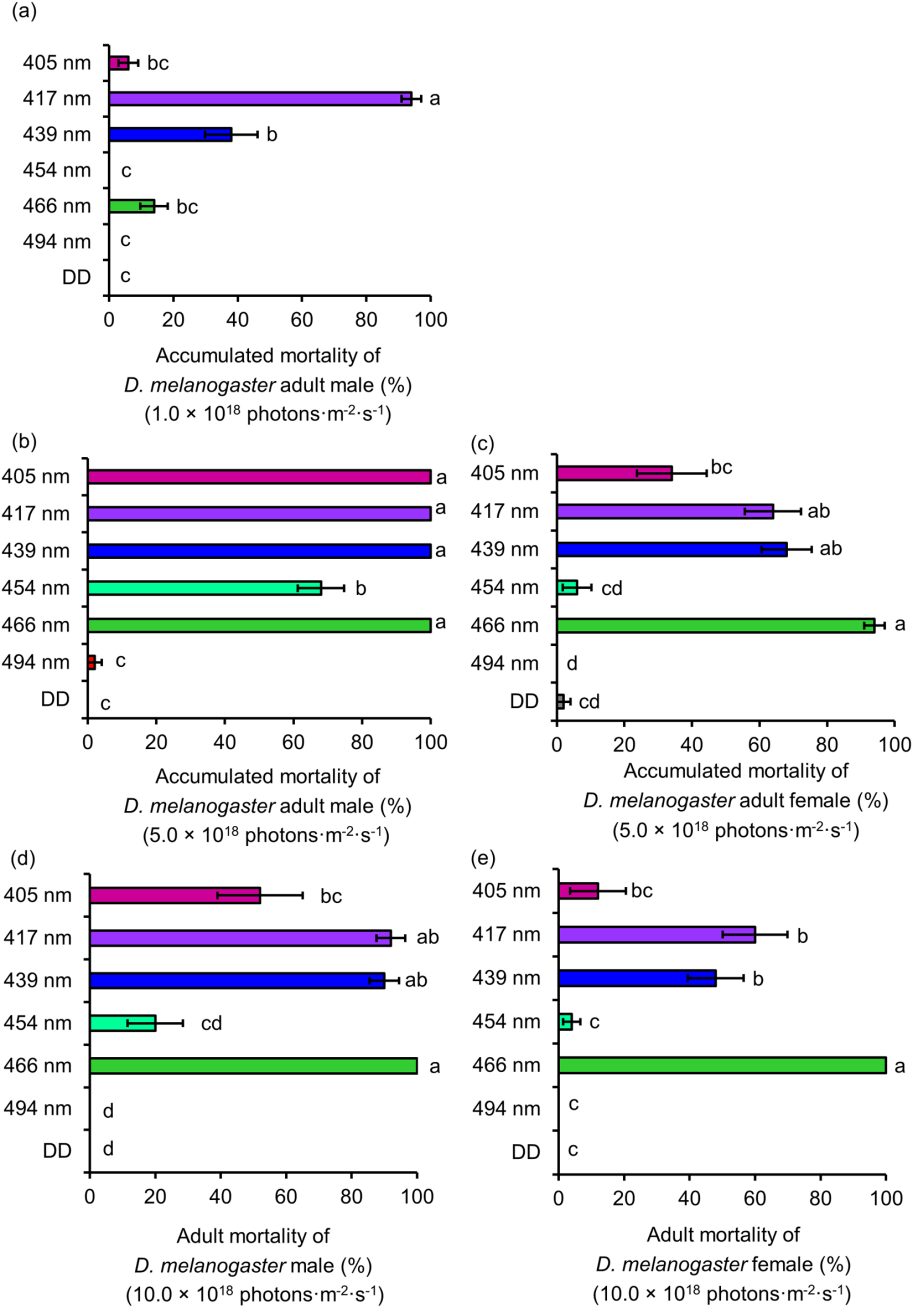
Lethal effect of different wavelengths of blue light on *D*. *melanogaster* adults. (a) Accumulated male mortality at 1.0 × 10^18^ photons·m^-2^·s^-1^ after 12 days (mean ± standard error). (b) Accumulated male mortality at 5.0 × 10^18^ photons·m^-2^·s^-1^ after 6 days (mean ± standard error). (c) Accumulated female mortality at 5.0 × 10^18^ photons·m^-2^·s^-1^ after 6 days (mean ± standard error). (d) Male mortality at 10.0 × 10^18^ photons·m^-2^·s^-1^ after 3 days (mean ± standard error). (e) Female mortality at 10.0 × 10^18^ photons·m^-2^·s^-1^ after 3 days (mean ± standard error). Different lowercase letters next to bars indicate significant differences (Steel-Dwass test, *P* < 0.05). DD: 24 h dark conditions.

To compare differences in male mortality among wavelengths after 12 days of irradiation, multiple comparisons were carried out (Fig 6a).

Mortality was significantly higher at 417 and 439 nm compared to DD; however, none of the other wavelengths were significantly different to DD. In contrast to males, no lethal effects were detected on females at any wavelength with 1.0 × 10^18^ photons·m^-2^·s^-1^ (Fig 5b). On day 12, the highest mortality of females was only 6% at 439 nm.

At 5.0 × 10^18^ photons·m^-2^·s^-1^, no wavelength exhibited any lethal effect on day 3, for both males and females (Fig 5c and 5d). However, on day 6 of irradiation, all wavelengths, other than 494 nm, showed lethal effects on males, with the highest mortality (100%) being recorded at 405, 417, 439, and 466 nm. On day 9, mortality of more than 92% of males was recorded for all tested wavelengths (Fig 5c). The lethal effects on females appeared later than those on males. Mortality of more than 90% of females was recorded for all wavelengths on day 12 (Fig 5d). To compare mortality between wavelengths, multiple comparisons were performed on day 6 of irradiation, which was when the greatest difference that occurred among wavelengths. Compared to DD, male mortality was significantly higher at all wavelengths, other than 494 nm (Fig 6b). However, compared to DD, significantly higher female mortality was recorded at 417, 439, and 466 nm. The highest female mortality was 94% at 466 nm, followed by 68% at 439 nm, 64% at 417 nm, and 34% at 405 nm. Compared to DD, there was no significant difference at 405, 454, and 494 nm on females (Fig 6c).

At 10.0 × 10^18^ photons·m^-2^·s^-1^, wavelengths of 417, 439, and 466 nm showed particularly high mortality on males on day 3, with 92%, 90%, and 100% mortality, respectively (Fig 5e). On day 6, all wavelengths showed extremely high mortality on males. The lowest mortality was 98% (494 nm), while all other wavelengths showed 100% mortality. On day 9, all tested males died. For females, the wavelength of 466 nm showed the highest mortality (100%) on day 3. However, the mortality of females at all other wavelengths was below 60%. On day 6, female mortality at all wavelengths, other than 494 nm, was over 90%. On day 9, all tested females died (Fig 5f). Multiple comparisons of mortality were made on day 3. Male mortality at 405, 417, 439, and 466 nm was significantly higher than that under DD (Fig 6d). Female mortality at 417, 439, and 466 nm was significantly higher than that under DD (Fig 6e).

### H_2_O_2_ generation by irradiating blue light on *D*. *melanogaster* pupae

**Fig 7:**
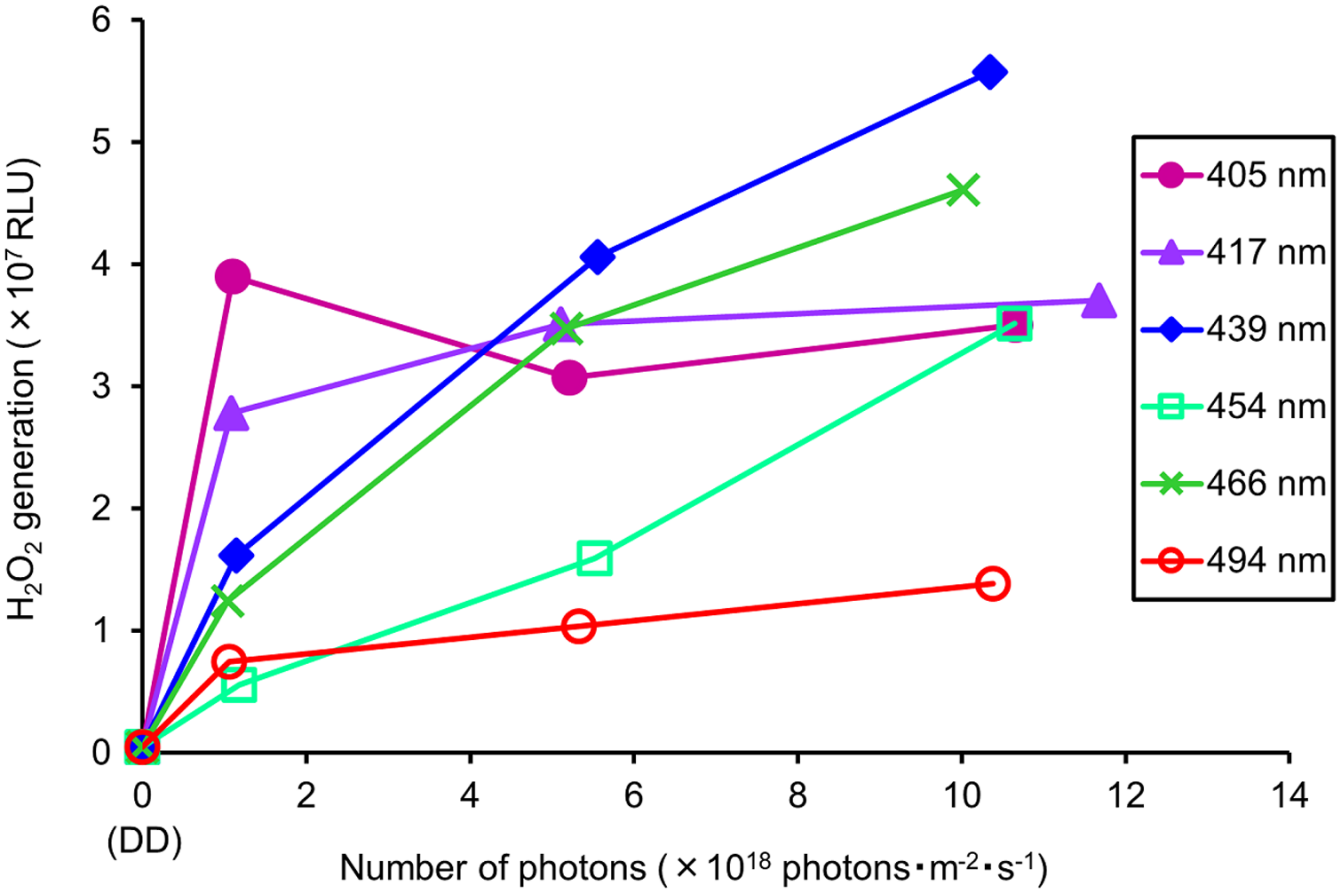
Dose-response relationship between light wavelength and H_2_O_2_ generation. RLU indicates the amount of luminescence. DD: 24 h dark conditions.

We investigated the amount of H_2_O_2_ that was generated in pupae irradiated with each light dose and wavelength. Thirty replications were performed (Fig 7).

H_2_O_2_ generation by irradiating blue light was showed. RLU increased as the number of photons increased. At 1.0 × 10^18^ photons·m^-2^·s^-1^, 405 nm showed the highest RLU (3.9 × 10^7^). At 5.0 × 10^18^ photons·m^-2^·s^-1^, 439 nm showed the highest RLU (4.06 × 10^7^). At 10.0 × 10^18^ photons·m^-2^·s^-1^, 439 nm showed the highest RLU (5.58 × 10^7^).

**Fig 8:**
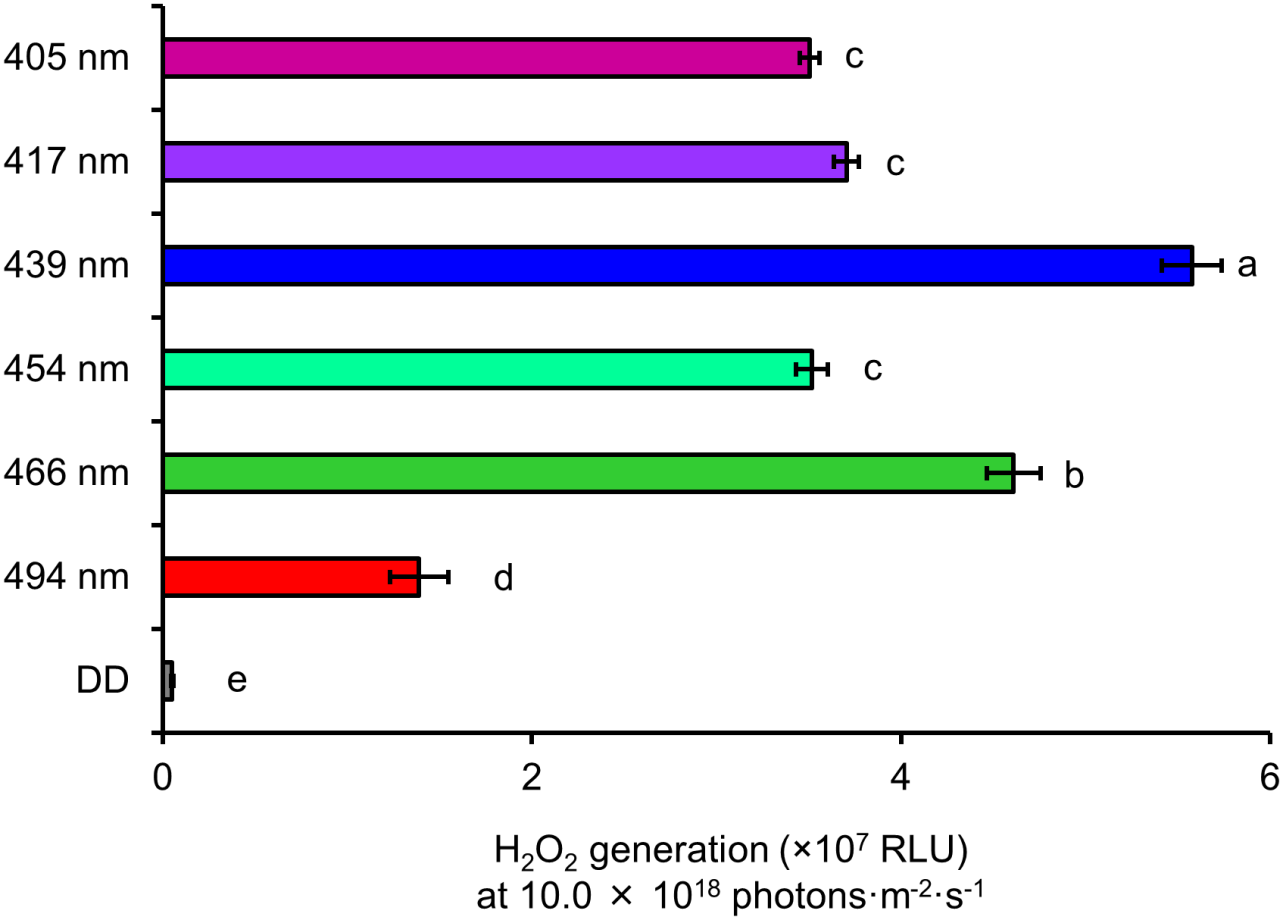
Comparison of H_2_O_2_ generation among the wavelengths of blue light at 10.0 × 10^18^ photons·m^-2^·s^-1^. Data are mean ± standard error. Different lowercase letters next to bars indicate significant differences (Tukey’s test, *P* < 0.01). RLU indicates the amount of luminescence. DD: 24 h dark conditions.

To compare differences in the generation of H_2_O_2_ between wavelengths, multiple comparisons were carried out at 10.0 × 10^18^ photons·m^-2^·s^-1^ (Fig 8).

The RLU showed that 405–466 nm was significantly higher than 494 nm. The wavelength of 439 nm had the highest RLU.

## Discussion

This study confirmed that blue light is lethal to all growth stages of *D*. *melanogaster*.

The inner space of the Petri dish did not register temperatures that would have affected the survival at any of the developmental stages in any of the irradiation treatments (S6–S9 Tables). In addition, the rise in mortality did not always correspond to a rise in temperature. Thus, we concluded that a rise in temperature caused by light irradiation did not influence the lethal effect.

We showed that the most toxic wavelength differed at different developmental stages. Morphological changes corresponding to metamorphosis might cause these differences in the responsiveness to light by the different developmental stages.

Shorter wavelengths were more harmful to eggs (Fig 1). Eggs were assumed to be damaged by light with a shorter wavelength because this wavelength contains more energy. However, lower mortality was detected at 439 nm compared to 417 and 454 nm. Certain structures might block the transmission of 439 nm light into the *D*. *melanogaster* body. Although the detailed mechanism remains unclear, embryo development might be inhibited by the energy of blue light.

The lethal effects of blue light were different on larvae compared to the eggs. There was no variation in the lethal effects of the 405 to 466 nm wavelengths on larvae (Fig 2). These contrasting results that were recorded for eggs and larvae might be caused by the chromophore and larval photoreceptor. For instance, certain chromophores that absorb light of 439 nm (for example, cytochrome c oxidase [22]) might exist in eggs and larvae. To prevent extensive damage by 439 nm light through the chromophore, specific structures of the egg shell might block the transmission of 439 nm light. Therefore, egg mortality at 439 nm was lower than that at 417 and 454 nm (Fig 1). In comparison, larvae do not have egg shells, but they do have photoreceptors, such as the Bolwig organ and class IV neuron [23,24]. The larval body is damaged by 439 nm light through the chromophore and by other wavelengths of blue light through the photoreceptors. Therefore, the mortality of larvae was consistent from 405 to 466 nm (Fig 2). To verify this hypothesis, further research about the chromophore and photoreceptor is needed.

The irradiation tests on pupae showed that mortality greatly decreases as pupal growth progresses (Fig 3a). In insects that exhibit holometabolism, such as *D*. *melanogaster*, the degeneration and disappearance of larval tissues and the formation of adult tissues occur during the pupal period [21]. Thus, the early stages of the pupal development might be more susceptible to damage than the latter phases. The lethal effects were divided between two groups in the experiments on P2–4 and P5 (Fig 3). Wavelengths from 417 nm to 466 nm showed strong lethal effects, whereas wavelengths of 405 and 494 nm produced no lethal effect. These results contrast with those of our previous study [20], in which a wavelength of 467 nm was the most lethal to *D*. *melanogaster* pupae, followed by 440 nm (bimodal peak), while 456 nm was not lethal. This difference might be due to the use of different experimental conditions. In our previous study, pupae were irradiated with 3.0 × 10^18^ photons·m^-2^·s^-1^ for 7 days. In contrast, pupae were irradiated with 10.0 × 10^18^ photons·m^-2^·s^-1^ for 24 h in the current study (as shown in Fig 3). Because the light was strong, mortality at each wavelength was higher, which might mask the bimodal peak. To verify this suggestion, we reduced the number of photons to 8.0 or 9.0 × 10^18^ photons·m^-2^·s^-1^ (Fig 4). The bimodal peak was detected when P5 were irradiated with 8.0 × 10^18^ photons·m^-2^·s^-1^; however, mortality at 439 nm was much lower compared to that recorded in the previous study (Fig 4b). The bimodal peak was clearly visible at 9.0 × 10^18^ photons·m^-2^·s^-1^ (Fig 4c and 4d). However, this peak did not correspond with that obtained in the previous study, as mortality was similar at 439 nm and 417 nm. Thus, the lethal effect of pupae might be influenced by a combination of the number of photons and the length of irradiation.

In the irradiation test for each wavelength of blue light on adults, the effective wavelength differed with the number of photons and sex. At 1.0 × 10^18^ photons·m^-2^·s^-1^, 417 nm caused the highest mortality to males. In contrast, this wavelength had no lethal effect on females. At 5.0 × 10^18^ photons·m^-2^·s^-1^, wavelengths of 405, 417, 439, and 466 nm had the strongest lethal effect on males. The effective wavelength was 466 nm on females. At 10.0 × 10^18^ photons·m^-2^·s^-1^, 466 nm caused the highest mortality to both sexes (Figs 5 and 6). These different susceptibilities between sex might due to differences in longevity. In our previous study, we showed that irradiation with 467 nm shortened adult longevity of *D*. *melanogaster* according to the number of photons [20]. Average longevity was 57.6 days (control), 19.4 days (1.0 × 10^18^ photons·m^-2^·s^-1^), 5.2 days (5.0 × 10^18^ photons·m^-2^·s^-1^), and 2.95 days (10 × 10^18^ photons·m^-2^·s^-1^). However, adult sex was not distinguished when calculating this average longevity. Thus, we recalculated the average longevity of each sex. Under control conditions, male longevity was 55.9 days and female longevity was 59.3 days. At 1.0 × 10^18^ photons·m^-2^·s^-1^, male longevity was 18.5 days and female longevity was 20.3 days. At 5.0 × 10^18^ photons·m^-2^·s^-1^, male longevity was 4.9 days and female longevity was 5.5 days. At 10 × 10^18^ photons·m^-2^·s^-1^, male longevity was 2.9 days and female longevity was 3.0 days. These results suggest that female longevity is longer than that of males. Because adult females have naturally longer longevity than adult males, the lethal effect of light irradiation might not be visible when using 1.0 × 10^18^ photons·m^-2^·s^-1^ for 12 days (Fig 5a and 5b). In our previous study, we showed that blue light irradiation affects both adult longevity and the oviposition ability of *D*. *melanogaster* [20]. Adult females irradiated with 467 nm at 1.0 × 10^18^ photons·m^-2^·s^-1^ laid much fewer eggs than non-irradiated (control condition) females. This result suggests that blue light irradiation could suppress the next generation.

As observed with pupae, damage might differ between protracted irradiation with weak light versus short irradiation with strong light. In addition, the lethal effects of blue light on adults were similar to those recorded on pupae (Figs 4 and 6). For instance, the results presented in Fig 4c and 4d are very similar to those presented in Fig 6c, 6d and 6e. We found that a wavelength of 466 nm was the most effective, followed by 417 and 439 nm. No lethal effect was detected at 454 nm. Therefore, sensitivity to blue light shifts from larval type to adult type during the prepupa stage (P2–4).

Our results clearly showed that effective wavelengths differ with respect to the growth stage of the insect. In addition, the results of our previous study demonstrated that the effective wavelengths that had a lethal effect were species-specific [20]. Therefore, species-specific and growth stage-specific photoreceptive parts might be associated with the lethal effect of blue light. In addition, reactive oxygen species (ROS) might contribute to damage caused by blue light irradiation. For instance, Kuse et al. (2014) reported that reactive oxygen is generated when blue light is irradiated on mouse cultured retinal cells [25]. Suzuki et al. (2012) reported that oxidized phospholipids in the mouse retina are induced by irradiation with blue light LED (light- emitting diode) [26]. Based on this information, we hypothesized the mechanism of the lethal effect of blue light on insects. Specifically, we suggest that when insects are irradiated with specific wavelengths of blue light, light energy is absorbed by species-specific or growth stage-specific parts (organs or tissues). In these organs and tissues, ROS are generated by absorbed light energy. The insect body is damaged by ROS, which causes the lethal effect. In fact, the level of hydrogen peroxide (H_2_O_2_, a kind of ROS) in the pupae of *D*. *melanogaster* (P5) rose after irradiating them with blue light. The level of H_2_O_2_ rose as the number of photons increased at all tested wavelengths (Fig 7). Furthermore, the level of H_2_O_2_ generation was wavelength-specific. At 10.0 × 10^18^ photons·m^-2^·s^-1^, wavelengths of 439 and 466 nm generated the highest amounts of H_2_O_2_. Wavelengths of 405, 417, and 454 nm also generated high amounts of H_2_O_2_. The wavelength of 494 nm generated the lowest amount of H_2_O_2_ (Fig 8). This specificity was similar to the wavelength-specific lethal effect on P5, which was irradiated with 10.0 × 10^18^ photons·m^-2^·s^-1^ (Fig 3c). When wavelengths were shorter than 494 nm, both the lethal effect and amount of H_2_O_2_ were high. In contrast, mortality and H_2_O_2_ levels were low at long wavelengths (494 nm). Therefore, ROS generation in the insect body and the lethal effect of blue light on insects might be related. A preliminary experiment of our laboratory showed that proliferation of cultured *D*. *melanogaster* cells was inhibited by irradiation with blue light. Thus, blue light irradiation might directly damage insect cells and cause the lethal effects. Thus, some sort of endogenous factor in cell could relate the lethal effect. In addition, a recent report showed that blue light exposure decreases melanin contents and impairs immune function of *Bactrocera dorsalis* [27]. These finding are expected to contribute toward clarifying the mechanism of the lethal effect of blue light on insects.

This study showed that the effective wavelength of the lethal effects of blue light varies with the growth stage of insects. This finding is important toward providing baseline information for establishing pest control methods using light irradiation. Effective pest control could be realized by clarifying the most lethal wavelength of the targeted growth stage. For example, the eggs and pupae could be targeted because these stages do not move. Adult insects that live in limited spaces could also be targeted, such as sanitary insect pests or stored grain insect pests. Our group is investigating the relationship between the lethal effects of blue light and developmental stages for over 10 insect species, such as *Galerucella grisescens*, *Clogmia albipunctatus*, *Liriomyza sativae*, and so on. For example, the eggs of *Galerucella grisescens*, the strawberry leaf beetle, are killed by irradiation at 438 nm; however, the pupae are not killed by this wavelength [28]. This previous report [28] only compared eggs and pupae, but the results suggest that the photosensitivity of other holometabolous insects might also change, depending on the stage of development. In comparison, our preliminary experiments suggest that the effective wavelength of certain insects does not change with development, as observed for *Culex pipiens molestus* (unpublished). Whether the effective wavelength changes might depend on the species of insect. Thus, to use blue light irradiation for pest control, we must first identify the effective wavelengths of each developmental stage for each targeted species.

In conclusion, the findings of this study could contribute toward advancing pest control techniques, because they clarify how the lethal effects of light are caused. Furthermore, this study provides important information on insect photobiology.

## Supporting information

S1 Table Actual measurement values of the number of photons in the egg irradiation experiment. Data are the mean ± standard error of each five measurements before and after the experiment.
(DOCX)

S2 Table Actual measurement values of the number of photons in the larva irradiation experiment. Data are the mean ± standard error of each five measurements before and after the experiment.
(DOCX)

S3 Table Actual measurement values of the number of photons in the pupa irradiation experiment. Data are the mean ± standard error of each five measurements before and after the experiment.
(DOCX)

S4 Table Actual measurement values of the number of photons in the adult irradiation experiment. Data are the mean ± standard error of each five measurements before and after the experiment.
(DOCX)

S5 Table Actual measurement values of the number of photons in the H_2_O_2_ generation experiment. Data are the mean ± standard error of each five measurements before and after the experiment.
(DOCX)

S6 Table Temperature of the inner space of the Petri dishes in the egg experiment. ^a^ Data are the mean of each five measurements before and after the experiment. ^b^ Data are the mean ± standard error of the 24 h period during irradiation.
(DOCX)

S7 Table Temperature of the inner space of the Petri dishes in the larva experiment. ^a^ Data are the mean of each five measurements before and after the experiment. ^b^ Data are the mean ± standard error of the 24 h period during irradiation.
(DOCX)

S8 Table Temperature of the inner space of the Petri dishes in the pupa experiment. ^a^ Data are the mean of each five measurements before and after the experiment. ^b^ Data are the mean ± standard error of the 24 h period during irradiation.
(DOCX)

S9 Table Temperature of the inner space of the Petri dishes in the adult experiment. ^a^ Data are the mean of each five measurements before and after the experiment. ^b^ Data are the mean ± standard error of the 24 h period during irradiation.
(DOCX)

S1 Fig Emission spectra of LED lighting units used for the experiments. (TIF)

S2 Fig Photographs of each growth stage of *Drosophila melanogaster* pupae. Puparia of P5–P14 were removed to clearly display pupal growth.
(TIF)

## References

[1] Cowan T, Gries G. Ultraviolet and violet light: attractive orientation cues for the Indian meal moth, *Plodia interpunctella*. Entomol Exp Appl. 2009;131: 148–158.

[2] Bickford ED. Biological lighting; 1964. Preprint. I. E. S. Nat. Tech. Conf., Preprint No. 2, pp. 2–3.

[3] Harada T. Effects of photoperiod and temperature on phototaxis in a water strider, *Gerris paludum insularis* (Motschulsky). J Insect Physiol. 1991;37: 27–34.

[4] Arbogast RT, Flaherty BR. Light responses of *Tribolium castaneum* and *Tribolium confusum* (Coleoptera, Tenebrionidae): variation with age and sex. J Stored Prod Res. 1973;9: 31–35.

[5] Yang EC, Lee DW, Wu WY. Action spectra of phototactic responses of flea beetle, *Phyllotreta striolata*. Physiol Entomol. 2003;28: 362–367.

[6] Matsumura M. The current status of occurrence and forecasting system of rice planthoppers in Japan. J Asia-Pacific Entomol. 2001;4: 195–199.

[7] Shimoda M, Honda K. Insect reactions to light and its applications to pest management. Appl Entomol Zool. 2013;48: 413–421.

[8] Meyer-Rochow VB. Fine structural changes in dark-light adaptation in relation to unit studies of an insect compound eye with a crustacean-like rhabdom. J Insect Physiol. 1974;20: 573–589. 4819574 10.1016/0022-1910(74)90164-4

[9] Nomura K, Oya S, Watanabe I, Kawamura H. Studies on orchard illumination against fruit-piercing moths. I. Analysis of illumination effects, and influence of light elements on moths’ activities. Jpn J Appl Entomol Zool. 1965;9: 179–186. Japanese with English summary.

[10] Nomura K. Some considerations on the effect of orchard illumination against fruit-piercing moths. Tech Bull Fac Hort Chiba Univ. 1966;14: 27–34. Japanese with English summary.

[11] Goodman LJ. The role of certain optomotor reactions in regulating stability in the rolling plane during flight in the desert locust, *Schistocerca gregaria*. J Exp Biol. 1965;43: 385–407.

[12] Simmons AM, Kousik CS, Levi A. Combining reflective mulch and host plant resistance for sweet potato whitefly (Hemiptera: Aleyrodidae) management in watermelon. Crop Prot. 2010;29: 898–902.

[13] Mishiro K, Arai T, Ohira Y. Effect of reflective mulching on migratory flight to citrus orchard in two stinkbugs, *Plautia crossota stali* and *Glaucias subpunctatus*. Bull Natl Inst Fruit Tree Sci. 2009;9: 23–30. Japanese with English summary.

[14] Ghanem I, Shamma M. Effect of non-ionizing radiation (UVC) on the development of *Trogoderma granarium* Everts. J Stored Prod Res. 2007;43: 362–366.

[15] Okamoto K. Test for cockroach control with UV radiation 1. Entrance of cockroaches into the UV radiation field of germicidal lamps. Jpn J Sanit Zool. 1989;40: 259–267.

[16] Okamoto K. The lethal effect of UV radiation on the adult German cockroach 1. Difference in the lethal effect by irradiation regimes. Jpn J Sanit Zool. 1992;43: 235–241.

[17] Cohen SH, Sousa JA, Roach JF. Effects of UV irradiation on nymphs of five species of cockroaches. J Econ Entomol. 1973;66: 859–862. 10.1093/jee/68.5.687 1184821

[18] Gingrich JB. Ultraviolet-induced histological and histochemical changes in the integument of newly molted American cockroaches, *Periplaneta americana* (Dictyoptera: Blattaria: Blattidae). Can J Zool. 1975;53: 154–159. 1116069 10.1139/z75-018

[19] Suzuki T, Yoshioka Y, Tsarsitalidou O, Ntalia V, Ohno S, Ohyama K, et al An LED-based UV-B irradiation system for tiny organisms: System description and demonstration experiment to determine the hatchability of eggs from four *Tetranychus* spider mite species from Okinawa. J Insect Physiol. 2014;62: 1–10. doi: 10.1016/j.jinsphys.2014.01.005 24462572 10.1016/j.jinsphys.2014.01.005

[20] Hori M, Shibuya K, Sato M, Saito Y. Lethal effects of short-wavelength visible light on insects. Sci Rep. 2014;4 http://www.nature.com/articles/srep07383. 10.1038/srep07383 PMC4260232 25488603

[21] Bainbridge SP, Bownes M. Staging the metamorphosis of *Drosophila melanogaster*. J Embryol Exp Morph. 1981;66: 57–80. 6802923

[22] Lubart R, Wollman Y, Friedmann H, Rochkind S, Laulicht I. Effects of visible and near-infrared lasers on cell cultures. J Photochem Photobiol B. 1992;12: 305–310. 1321905 10.1016/1011-1344(92)85032-p

[23] Sprecher SG, Desplan C. Switch of rhodopsin expression in terminally differentiated *Drosophila* sensory neurons. Nature. 2008;454: 533–537. doi: 10.1038/nature07062 18594514 10.1038/nature07062 PMC2750042

[24] Xiang Y, Yuan Q, Vogt N, Looger LL, Jan LY, Jan YN. Light-avoidance-mediating photoreceptors tile the *Drosophila* larval body wall. Nature. 2010;468: 921–926. doi: 10.1038/nature09576 21068723 10.1038/nature09576 PMC3026603

[25] Kuse Y, Ogawa K, Tsuruma K, Shimazawa M, Hara H. Damage of photoreceptor-derived cells in culture induced by light emitting diode-derived blue light. Sci Rep. 2014;4 http://www.nature.com/articles/srep05223 10.1038/srep05223 PMC4048889 24909301

[26] Suzuki M, Tsujikawa M, Itabe H, Du ZJ, Xie P, Matsumura N, et al Chronic photo-oxidative stress and subsequent MCP-1 activation as causative factors for age-related macular degeneration. J Cell Sci. 2012;125: 2407–2415. doi: 10.1242/jcs.097683 22357958 10.1242/jcs.097683 PMC3383257

[27] Tariq K, Noor M, Hori M, Ali A, Hussain A, Peng W, et al Blue light-induced immunosuppression in *Bactrocera dorsalis* adults, as a carryover effect of larval exposure. Bull Entomol Res. 2017;1–8. 10.1017/S0007485317000438 28485267

[28] Hori M, Suzuki A. Lethal effect of blue light on strawberry leaf beetle, *Galerucella grisescens* (Coleoptera: Chrysomelidae). Sci Rep. 2017;7 http://www.nature.com/articles/s41598-017-03017-z 10.1038/s41598-017-03017-z PMC5457428 28578425

