# Supplementary material for: Toxic wavelength of blue light changes as insects grow
Source: PLoS One. 2018 Jun 19;13(6):e0199266. doi: 10.1371/journal.pone.0199266 (PMC6007831; doi:10.1371/journal.pone.0199266)
Supplement: S1 Table — Data are the mean ± standard error of each five measurements before and after the experiment. (DOCX) [file pone.0199266.s001.docx]

| Wavelength  (nm) | Number of photons  (× 10^18^ photons･m^-2^･s^-1^) | Wavelength  (nm) | Number of photons  (× 10^18^ photons･m^-2^･s^-1^) |
| --- | --- | --- | --- |
| 405 | 2.13 ± 0.04 | 454 | 1.78 ± 0.08 |
|  | 3.06 ± 0.01 |  | 3.03 ± 0.004 |
|  | 4.22 ± 0.06 |  | 4.38 ± 0.12 |
|  | 5.14 ± 0.05 |  | 5.11 ± 0.02 |
|  | 9.33 ± 0.23 |  | 9.47 ± 0.18 |
| 417 | 1.96 ± 0.02 | 466 | 2.94 ± 0.02 |
|  | 3.49 ± 0.16 |  | 4.05 ± 0.01 |
|  | 4.38 ± 0.13 |  | 5.29 ± 0.07 |
|  | 5.02 ± 0.004 |  | 9.75 ± 0.10 |
|  | 10.46 ± 0.14 | 494 | 4.51 ± 0.16 |
| 439 | 1.76 ± 0.08 |  | 8.44 ± 0.17 |
|  | 3.03 ± 0.04 |  | 9.55 ± 0.09 |
|  | 3.88 ± 0.04 |  |  |
|  | 5.37 ± 0.12 |  |  |
|  | 8.73 ± 0.13 |  |  |
