# Supplementary material for: Toxic wavelength of blue light changes as insects grow
Source: PLoS One. 2018 Jun 19;13(6):e0199266. doi: 10.1371/journal.pone.0199266 (PMC6007831; doi:10.1371/journal.pone.0199266)
Supplement: S2 Table — Data are the mean ± standard error of each five measurements before and after the experiment. (DOCX) [file pone.0199266.s002.docx]

| Wavelength  (nm) | Number of photons  (× 10^18^ photons･m^-2^･s^-1^) | Wavelength  (nm) | Number of photons  (× 10^18^ photons･m^-2^･s^-1^) |
| --- | --- | --- | --- |
| 405 | 2.98 ± 0.02 | 454 | 2.87 ± 0.01 |
|  | 4.67 ± 0.10 |  | 5.01 ± 0.005 |
|  | 7.07 ± 0.04 |  | 6.88 ± 0.03 |
|  | 9.33 ± 0.19 |  | 10.04 ± 0.06 |
| 417 | 2.86 ± 0.03 | 466 | 2.92 ± 0.002 |
|  | 4.78 ± 0.09 |  | 4.67 ± 0.11 |
|  | 7.49 ± 0.16 |  | 7.27 ± 0.13 |
|  | 9.58 ± 0.06 |  | 9.35 ± 0.17 |
| 439 | 3.17 ± 0.07 | 494 | 4.70 ± 0.10 |
|  | 5.04 ± 0.01 |  | 6.29 ± 0.23 |
|  | 6.56 ± 0.14 |  |  |
|  | 10.37 ± 0.15 |  |  |
