# Supplementary material for: Toxic wavelength of blue light changes as insects grow
Source: PLoS One. 2018 Jun 19;13(6):e0199266. doi: 10.1371/journal.pone.0199266 (PMC6007831; doi:10.1371/journal.pone.0199266)
Supplement: S3 Table — Data are the mean ± standard error of each five measurements before and after the experiment. (DOCX) [file pone.0199266.s003.docx]

| Wavelength  (nm) | Number of photons  (× 10^18^ photons･m^-2^･s^-1^) |
| --- | --- |
| 405 | 11.06 ± 0.32 |
| 417 | 8.16 ± 0.02 |
|  | 9.32 ± 0.03 |
|  | 10.83 ± 0.02 |
| 439 | 7.93 ± 0.09 |
|  | 9.30 ± 0.01 |
|  | 11.33 ± 0.02 |
| 454 | 8.15 ± 0.04 |
|  | 9.17 ± 0.04 |
|  | 10.63 ± 0.01 |
| 466 | 8.17 ± 0.01 |
|  | 9.04 ± 0.03 |
|  | 9.91 ± 0.04 |
| 494 | 10.67 ± 0.11 |
