# Supplementary material for: Toxic wavelength of blue light changes as insects grow
Source: PLoS One. 2018 Jun 19;13(6):e0199266. doi: 10.1371/journal.pone.0199266 (PMC6007831; doi:10.1371/journal.pone.0199266)
Supplement: S4 Table — Data are the mean ± standard error of each five measurements before and after the experiment. (DOCX) [file pone.0199266.s004.docx]

| Wavelength  (nm) | Number of photons  (× 10^18^ photons･m^-2^･s^-1^) |
| --- | --- |
| 405 | 1.04 ± 0.01 |
|  | 5.31 ± 0.02 |
|  | 9.60 ± 0.22 |
| 417 | 1.31 ± 0.07 |
|  | 5.30 ± 0.04 |
|  | 10.47 ± 0.06 |
| 439 | 1.19 ± 0.04 |
|  | 5.68 ± 0.20 |
|  | 9.66 ± 0.14 |
| 454 | 0.95 ± 0.03 |
|  | 5.23 ± 0.05 |
|  | 9.36 ± 0.30 |
| 466 | 1.21 ± 0.04 |
|  | 4.89 ± 0.07 |
|  | 10.47 ± 0.05 |
| 494 | 1.04 ± 0.002 |
|  | 5.48 ± 0.13 |
|  | 9.58 ± 0.07 |
