# Supplementary material for: Toxic wavelength of blue light changes as insects grow
Source: PLoS One. 2018 Jun 19;13(6):e0199266. doi: 10.1371/journal.pone.0199266 (PMC6007831; doi:10.1371/journal.pone.0199266)
Supplement: S5 Table — Data are the mean ± standard error of each five measurements before and after the experiment. (DOCX) [file pone.0199266.s005.docx]

| Wavelength  (nm) | Number of photons  (× 10^18^ photons･m^-2^･s^-1^) |
| --- | --- |
| 405 | 1.10 ± 0.02 |
|  | 5.21 ± 0.01 |
|  | 10.65 ± 0.02 |
| 417 | 1.08 ± 0.005 |
|  | 5.10 ± 0.01 |
|  | 11.68 ± 0.38 |
| 439 | 1.15 ± 0.01 |
|  | 5.55 ± 0.14 |
|  | 10.35 ± 0.16 |
| 454 | 1.18 ± 0.01 |
|  | 5.52 ± 0.09 |
|  | 10.65 ± 0.03 |
| 466 | 1.04 ± 0.01 |
|  | 5.18 ± 0.01 |
|  | 10.01 ± 0.04 |
| 494 | 1.06 ± 0.02 |
|  | 5.33 ± 0.03 |
|  | 10.38 ± 0.11 |
