# Supplementary material for: Toxic wavelength of blue light changes as insects grow
Source: PLoS One. 2018 Jun 19;13(6):e0199266. doi: 10.1371/journal.pone.0199266 (PMC6007831; doi:10.1371/journal.pone.0199266)
Supplement: S6 Table — a Data are the mean of each five measurements before and after the experiment. b Data are the mean ± standard error of the 24 h period during irradiation. (DOCX) [file pone.0199266.s006.docx]

| Wavelength  (nm) | Number of photons ^a^  (× 10^18^ photons･m^-2^･s^-1^ ) | Temperature ^b^  (mean ± SE °C) |
| --- | --- | --- |
| 405 | 9.98 | 26.12 ± 0.01 |
| 417 | 10.83 | 26.04 ± 0.01 |
| 439 | 9.3 | 25.99 ± 0.005 |
| 454 | 10.16 | 24.54 ± 0.01 |
| 466 | 10.73 | 26.29 ± 0.02 |
| 494 | 10.08 | 25.06 ± 0.01 |
| DD | 0 | 24.78 ± 0.01 |
