# Supplementary material for: Toxic wavelength of blue light changes as insects grow
Source: PLoS One. 2018 Jun 19;13(6):e0199266. doi: 10.1371/journal.pone.0199266 (PMC6007831; doi:10.1371/journal.pone.0199266)
Supplement: S7 Table — a Data are the mean of each five measurements before and after the experiment. b Data are the mean ± standard error of the 24 h period during irradiation. (DOCX) [file pone.0199266.s007.docx]

| Wavelength  (nm) | Number of photons ^a^  (× 10^18^ photons･m^-2^･s^-1^ ) | Temperature ^b^  (mean ± SE °C) |
| --- | --- | --- |
| 405 | 10.15 | 26.46 ± 0.01 |
| 417 | 10.57 | 26.01 ± 0.01 |
| 439 | 10.84 | 26.55 ± 0.01 |
| 454 | 10.24 | 23.69 ± 0.02 |
| 466 | 10.38 | 26.56 ± 0.01 |
| 494 | 6.67 | 23.97 ± 0.01 |
| DD | 0 | 24.56 ± 0.01 |
