# Supplementary figures and images for: Toxic wavelength of blue light changes as insects grow
Source: PLoS One. 2018 Jun 19;13(6):e0199266. doi: 10.1371/journal.pone.0199266 (PMC6007831; doi:10.1371/journal.pone.0199266)

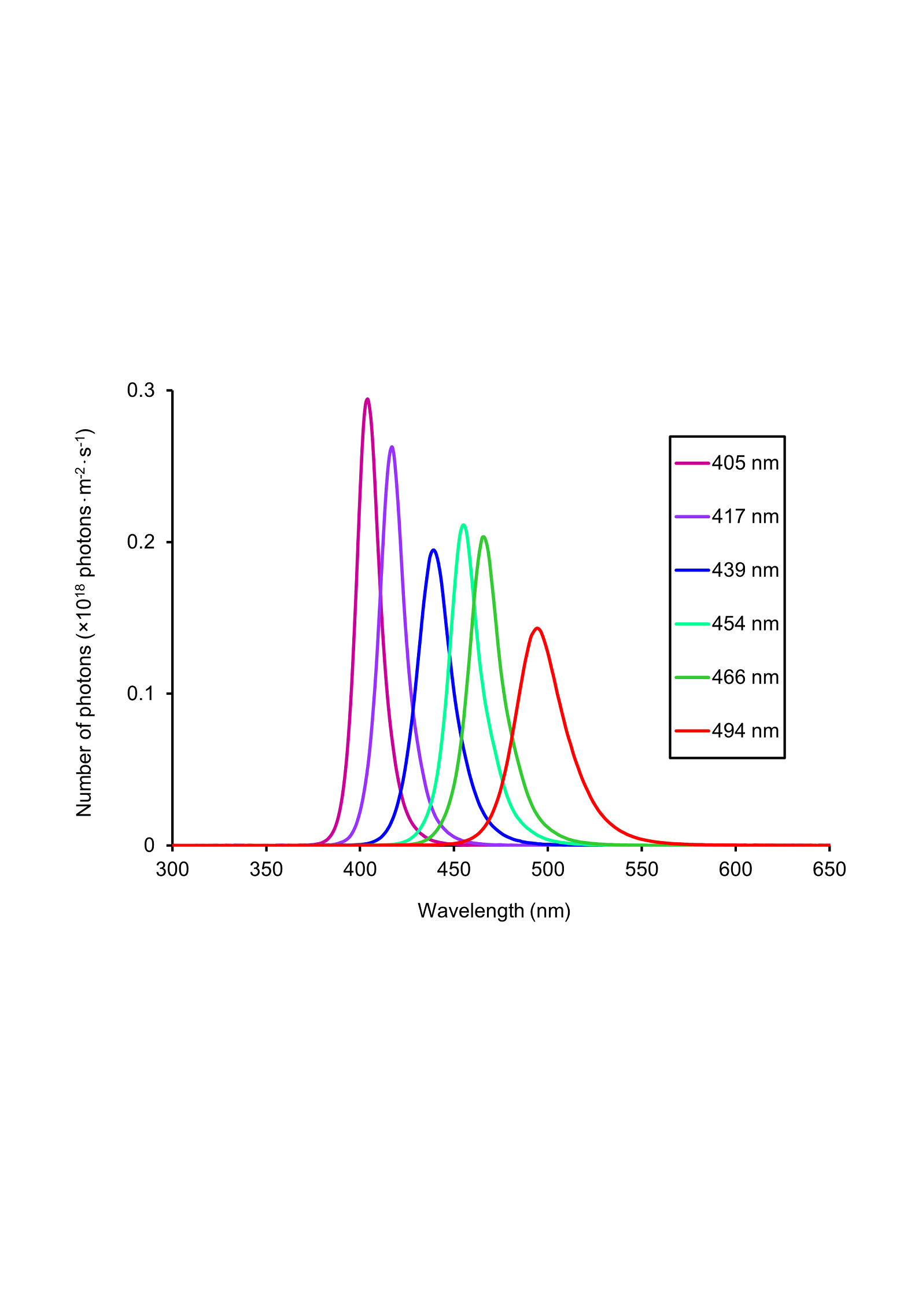

Supplement: S1 Fig — (TIF) [file pone.0199266.s010.tif]

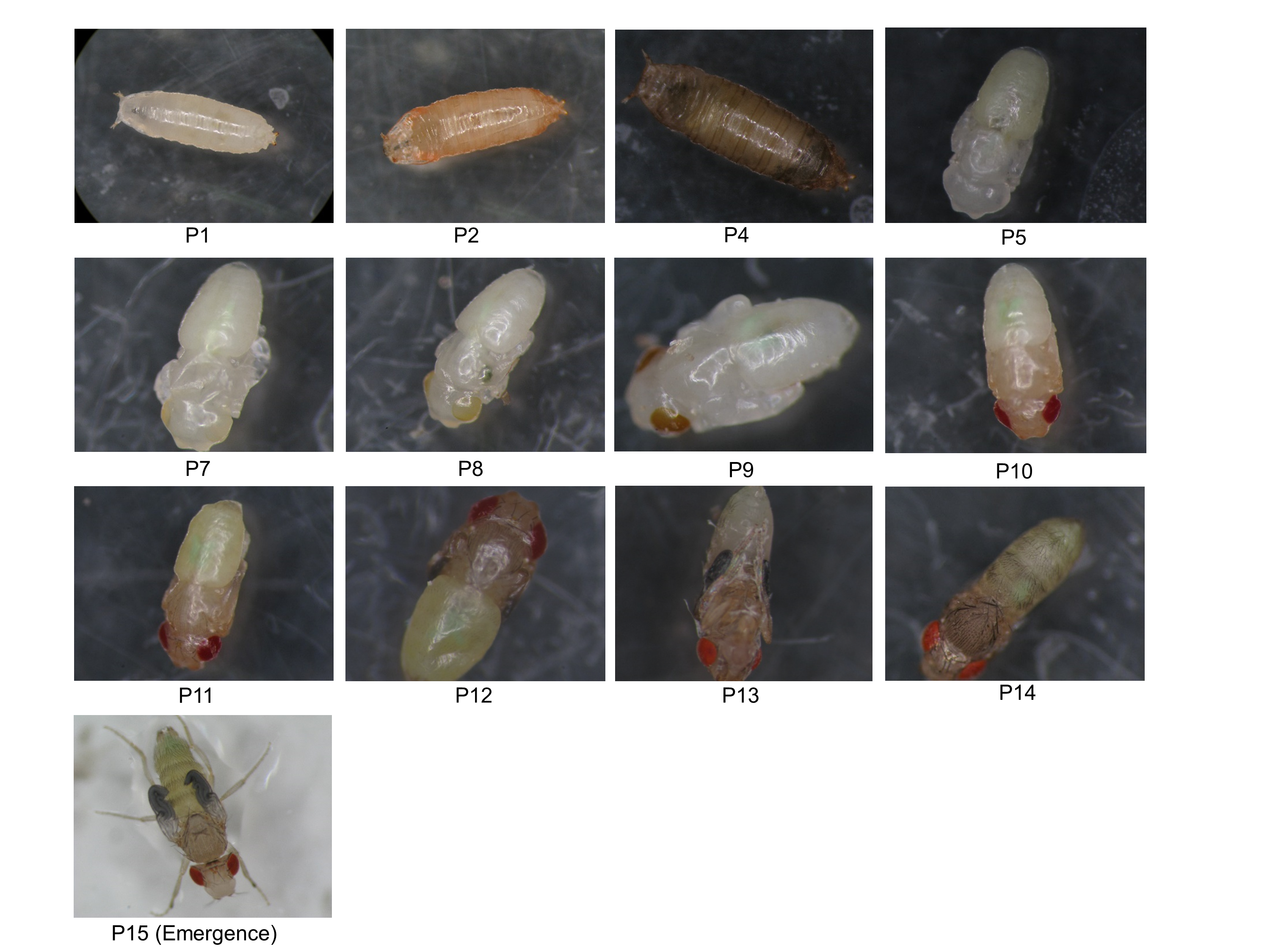

Supplement: S2 Fig — Puparia of P5–P14 were removed to clearly display pupal growth. (TIF) [file pone.0199266.s011.tif]
